# Zinc Toxicity and Iron-Sulfur Cluster Biogenesis in *Escherichia coli*

**DOI:** 10.1128/AEM.01967-18

**Published:** 2019-04-18

**Authors:** Jianghui Li, Xiaojun Ren, Bingqian Fan, Zhaoyang Huang, Wu Wang, Huaibin Zhou, Zhefeng Lou, Huangen Ding, Jianxin Lyu, Guoqiang Tan

**Affiliations:** a Laboratory of Molecular Medicine, Zhejiang Provincial Key Laboratory for Technology and Application of Model Organisms, Key Laboratory of Laboratory Medicine, Ministry of Education, China, School of Laboratory Medicine and Life Sciences, Wenzhou Medical University, Wenzhou, Zhejiang, China; b Zhejiang Provincial Key Laboratory of Medical Genetics, Key Laboratory of Laboratory Medicine, Ministry of Education, China, School of Laboratory Medicine and Life Sciences, Wenzhou Medical University, Wenzhou, Zhejiang, China; c Department of Biological Sciences, Louisiana State University, Baton Rouge, Louisiana, USA; Wageningen University

**Keywords:** IscA, IscU, ferredoxin, iron-sulfur proteins, zinc toxicity

## Abstract

While zinc is an essential trace metal in biology, excess zinc is toxic to organisms. Previous studies have shown that zinc toxicity is associated with disruption of the [4Fe-4S] clusters in various dehydratases in Escherichia coli. Here, we report that the intracellular zinc overload in E. coli cells inhibits iron-sulfur cluster biogenesis without affecting the preassembled iron-sulfur clusters in proteins. Among the housekeeping iron-sulfur cluster assembly proteins encoded by the gene cluster *iscSUA-hscBA-fdx-iscX* in E. coli cells, the scaffold IscU, the iron chaperone IscA, and ferredoxin have strong zinc binding activity in cells, suggesting that intracellular zinc overload inhibits iron-sulfur cluster biogenesis by binding to the iron-sulfur cluster assembly proteins. Mutations of the conserved cysteine residues to serine in IscA, IscU, or ferredoxin completely abolish the zinc binding activity of the proteins, indicating that zinc can compete with iron or iron-sulfur cluster binding in IscA, IscU, and ferredoxin and block iron-sulfur cluster biogenesis. Furthermore, intracellular zinc overload appears to emulate the slow-growth phenotype of the E. coli mutant cells with deletion of the iron-sulfur cluster assembly proteins IscU, IscA, and ferredoxin. Our results suggest that intracellular zinc overload inhibits iron-sulfur cluster biogenesis by targeting the iron-sulfur cluster assembly proteins IscU, IscA, and ferredoxin in E. coli cells.

**IMPORTANCE** Zinc toxicity has been implicated in causing various human diseases. High concentrations of zinc can also inhibit bacterial cell growth. However, the underlying mechanism has not been fully understood. Here, we report that zinc overload in Escherichia coli cells inhibits iron-sulfur cluster biogenesis by targeting specific iron-sulfur cluster assembly proteins. Because iron-sulfur proteins are involved in diverse physiological processes, the zinc-mediated inhibition of iron-sulfur cluster biogenesis could be largely responsible for the zinc-mediated cytotoxicity. Our finding provides new insights on how intracellular zinc overload may inhibit cellular functions in bacteria.

## Abstract (precis)

Zinc toxicity has been implicated in causing various human diseases. High concentrations of zinc can also inhibit bacterial cell growth. However, the underlying mechanism has not been fully understood. Here, we report that zinc overload in Escherichia coli cells inhibits iron-sulfur cluster biogenesis by targeting specific iron-sulfur cluster assembly proteins. Because iron-sulfur proteins are involved in diverse physiological processes, the zinc-mediated inhibition of iron-sulfur cluster biogenesis could be largely responsible for the zinc-mediated cytotoxicity. Our finding provides new insights on how intracellular zinc overload may inhibit cellular functions in bacteria.

## INTRODUCTION

As an essential trace element, zinc is vitally important for all living organisms (1). At least 300 enzymes in the metabolic pathways of sugars, lipids, proteins, and nucleic acids use zinc as a cofactor (2). Lack of zinc has been attributed to many human health complications, including growth retardation, poor appetite, and cell-mediated immune dysfunction (3). On the other hand, excess zinc is highly toxic to cells (4–6). For example, an elevated intracellular zinc content has been linked to Alzheimer’s disease (7) and Kufor-Rakeb syndrome (juvenile Parkinsonism) (8). In Escherichia coli cells, intracellular total zinc accumulates to about 0.2 mM when cells are grown in LB medium (9). Radioactive ^65^Zn-labeling studies (10) and proteomic analyses (11) have revealed a large number of putative zinc-binding proteins in E. coli cells. On the other hand, the addition of 2.5 mM ZnSO_4_ to LB medium (12) or 0.35 mM ZnSO_4_ to M9 minimum medium (13) completely inhibits E. coli cell growth. Because of the zinc-mediated inhibition of cell growth, zinc compounds have been developed as antibacterial agents and preservatives. Furthermore, host-mediated zinc toxicity to pathogenic bacteria has been extensively investigated (14–16). However, the molecular mechanism underlying the zinc-mediated cytotoxicity has not been fully understood.

Our previous studies have shown that topoisomerase I (17, 18) and its homolog YrdD (19) are iron and zinc binding proteins, and excess zinc can easily compete for iron binding in the proteins *in vivo* (17, 19). This suggests that zinc and iron may have similar binding sites in proteins.

In the past decade, several “zinc finger” proteins have been identified as iron-sulfur proteins. For example, the mitochondrial outer membrane protein mitoNEET (20) and the cleavage and polyadenylation specificity factor 30 (CPSF30) (21) have a zinc finger motif which hosts an iron-sulfur cluster. Since zinc and iron-sulfur cluster have similar ligand coordination in proteins, it has been proposed that zinc may compete for iron-sulfur center binding sites in proteins and disrupt iron-sulfur clusters in proteins (22–24). Since iron-sulfur proteins are involved in diverse physiological functions (25), excess zinc may affect multiple cellular functions by disrupting iron-sulfur clusters in proteins.

Iron-sulfur clusters are assembled by a group of dedicated proteins. In E. coli, there are two iron-sulfur cluster assembly systems encoded by the housekeeping *iscSUA-hscBA-fdx-iscX* gene cluster (26) and the inducible *sufABCDSE* gene cluster (22). Among the proteins encoded by *iscSUA-hscBA-fdx-iscX*, IscS is a cysteine desulfurase that provides sulfur for iron-sulfur cluster assembly (27). IscU is a scaffold protein that assembles iron-sulfur clusters (28) and transfers the transient clusters to target proteins (29, 30). IscA was thought to be an alternative scaffold (31). However, unlike the scaffold IscU, IscA has strong iron binding activity, and the iron center in IscA can be transferred to IscU for iron-sulfur cluster assembly (32–34). Thus, IscA is proposed as an iron chaperone for iron-sulfur cluster biogenesis. HscB and HscA are heat shock cognate proteins, which assist the iron-sulfur cluster transfer from IscU to target protein (35). Ferredoxin (Fdx) is a [2Fe-2S] cluster protein and may provide electrons for the iron-sulfur cluster assembly process (36). IscX has also been proposed as an iron donor for iron-sulfur cluster biogenesis (37). However, IscX has low iron binding affinity and interacts with IscS (38). The deletion of IscX does not significantly affect iron-sulfur proteins in E. coli cells (39). Therefore, the specific function of IscX remains to be defined.

In this study, we find that zinc overload in E. coli cells inhibits iron-sulfur cluster biogenesis without affecting the preassembled clusters in proteins. Additional studies reveal that zinc has strong interaction with the iron-sulfur cluster assembly proteins IscU, IscA, and ferredoxin, leading to inhibition of iron-sulfur cluster biogenesis in E. coli cells.

## RESULTS

### Zinc overload selectively inactivates iron-sulfur enzymes in E. coli cells.

In wild-type E. coli cells, the “free” intracellular zinc concentration is in the femtomolar range (9). Zinc homeostasis in E. coli cells is regulated primarily through a network of zinc influx and efflux pumps. The major zinc efflux system ZntA, a P-type ATPase transporter, is upregulated by the transcription factor ZntR when intracellular zinc concentration is high (40). The deletion of ZntA results in an E. coli strain that is hypersensitive to zinc (41). To explore the effect of intracellular zinc overload on iron-sulfur proteins in E. coli cells, we have constructed an E. coli mutant in which both the zinc efflux pump ZntA and the transcription factor ZntR were deleted. Table 1 shows that the deletion of ZntA and ZntR resulted in accumulation of intracellular zinc in E. coli cells grown in LB medium supplemented with 200 μM ZnSO_4_ under aerobic growth conditions. ZnSO_4_ at 200 μM was chosen, as it inhibited cell growth of the E. coli *zntA zntR* double mutant in LB medium by about 50% and did not significantly affect the cell growth of wild-type E. coli (see Fig. S1 in the supplemental material).

**TABLE 1:** Zinc content of whole cells after zinc treatment

Strain	Mean ± SD zinc content (μM) of whole cells (per 100 OD at 600 nm)
MC4100	21.6 ± 0.6
MC4100+Zn	24.4 ± 4.2
*zntA zntR* mutant	25.1 ± 0.4
*zntA zntR* mutant + Zn	59.4 ± 2.4

**FIG 1:**
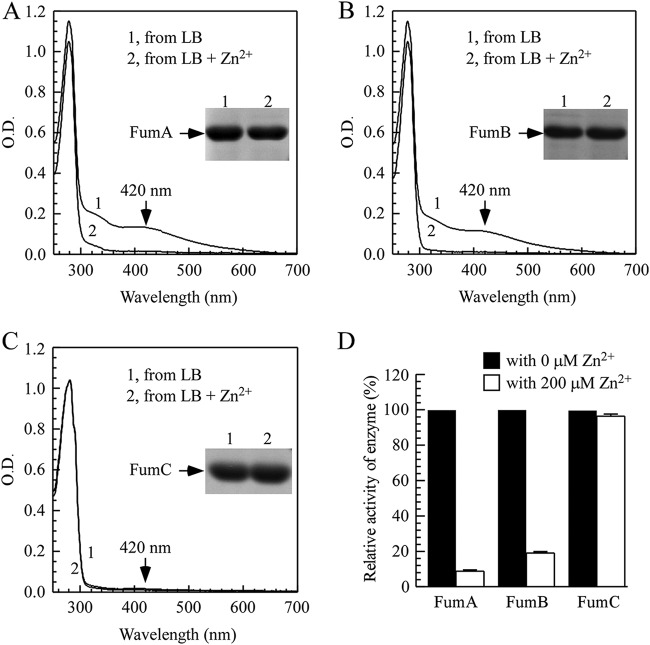
Zinc overload selectively inactivates iron-sulfur cluster containing fumarases by producing more apo forms. (A) UV-visible absorption spectra of recombinant fumarase A (FumA) proteins purified from *E. coli zntA zntR* double-mutant cells supplemented with 0 μM (spectrum 1) or 200 μM (spectrum 2) ZnSO_4_ in LB medium. (B) UV-visible absorption spectra of recombinant fumarase B (FumB) proteins purified from *E. coli zntA zntR* double-mutant cells supplemented with 0 μM (spectrum 1) or 200 μM (spectrum 2) ZnSO_4_ in LB medium. (C) UV-visible absorption spectra of recombinant fumarase C (FumC) proteins purified from *E. coli zntA zntR* double-mutant cells supplemented with 0 μM (spectrum 1) or 200 μM (spectrum 2) ZnSO_4_ in LB medium. The inset in panels A to C is a photograph of the SDS-PAGE gel of purified proteins. (D) The relative fumarase activity of purified proteins from panels A to C. The relative activity is representative of the percentage of fumarase activity with 200 μM ZnSO_4_ treatment in untreated samples. The results represent average ± standard deviation from three independent experiments.

To investigate the effect of zinc overload on iron-sulfur proteins in E. coli, we first utilized fumarases. There are three fumarases in E. coli, fumarase A and fumarase B, which require a [4Fe-4S] cluster for their catalytic activity (42); and fumarase C, which has no iron-sulfur clusters (43). Each fumarase was expressed in the E. coli *zntA zntR* mutant cells grown in LB medium supplemented with or without 200 μM ZnSO_4_ under aerobic conditions. Figure 1 shows that the addition of ZnSO_4_ (200 μM) to LB medium largely eliminated the iron-sulfur cluster content (Fig. 1A and B) and the enzyme activity (Fig. 1D) of fumarases A and B in the E. coli *zntA zntR* mutant cells. On the other hand, the same zinc treatment did not affect the enzyme activity of fumarase C in E. coli *zntA zntR* mutant cells (Fig. 1C and D). The results suggested that zinc overload in E. coli cells selectively inhibits iron-sulfur cluster-containing fumarases A and B without inhibiting fumarase C, which does not have iron-sulfur clusters.

### Effect of zinc overload on other iron-sulfur proteins in E. coli cells.

**FIG 2:**
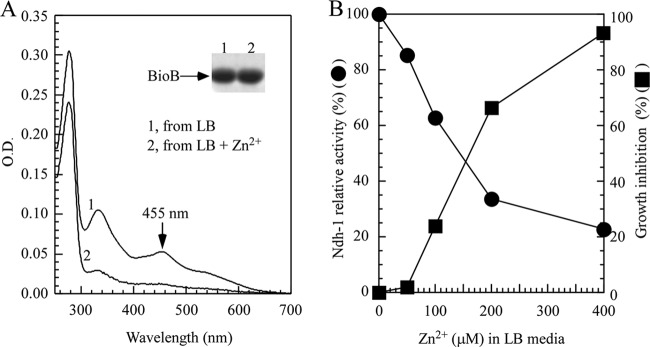
Excess zinc disrupts iron-sulfur cluster assembly in both [4Fe-4S] proteins and [2Fe-2S] proteins. (A) UV-visible absorption spectra of recombinant BioB purified from the *E. coli zntA zntR* double-mutant cells supplemented with 0 μM (spectrum 1) or 200 μM (spectrum 2) ZnSO_4_ in LB medium under aerobic growth conditions. (B) Correlation of the relative activity of NADH dehydrogenase I and cell growth inhibition. The relative dehydrogenase I activity and relative cell growth were defined as the percentage of the *E. coli zntA zntR* double-mutant cells in LB medium with ZnSO_4_ over that without ZnSO_4_. The relative cell growth inhibition rate was calculated by 100% minus the relative growth rate of the *E. coli zntA zntR* double-mutant cells. The relative dehydrogenase I activity (closed circles) and relative cell growth inhibition rate (closed squares) were plotted as a function of the ZnSO_4_ concentration in LB medium. The 100% cell growth represented the cell density (OD at 600 nm) of ∼3.0 after 5 h at 37°C in LB medium with aeration. The results were the most representative of three independent experiments.

To further explore the effects of zinc overload on iron-sulfur proteins, we used biotin synthase, which contains a [2Fe-2S] cluster and a [4Fe-4S] cluster (44). Biotin synthase (BioB) converts dethiobiotin into biotin by inserting a sulfur atom between C-6 and C-9 of dethiobiotin in an *S*-adenosylmethionine (SAM)-dependent reaction (45). In the experiments, recombinant BioB was expressed in the E. coli *zntA zntR* mutant cells grown in LB medium supplemented with or without 200 μM ZnSO_4_ under aerobic conditions. Figure 2A shows that the addition of ZnSO_4_ (200 μM) also decreased the iron-sulfur cluster contents of recombinant biotin synthase in the E. coli *zntA zntR* mutant cells.

We then analyzed the activity of the endogenous NADH dehydrogenase I, which contains multiple iron-sulfur clusters (46). Using deamino-NADH as a specific substrate for NADH dehydrogenase I (47), we found that the deamino-NADH oxidation rate of the E. coli *zntA zntR* mutant cells progressively decreased when the ZnSO_4_ concentration in LB medium was gradually increased from 0 to 400 μM (Fig. 2B). The cell growth of the E. coli *zntA zntR* mutant was also inhibited as the ZnSO_4_ concentration in LB medium was increased (Fig. 2B). Since the NADH dehydrogenase I contains multiple iron-sulfur clusters (48), the correlation between the decrease in the deamino-NADH oxidation rate and inhibition of cell growth by zinc in LB medium suggested that zinc toxicity could be closely associated with the inhibition of iron-sulfur proteins in E. coli cells.

### Zinc overload inhibits iron-sulfur cluster biogenesis in E. coli cells.

It was proposed that zinc may directly attack iron-sulfur clusters in proteins to produce the apo form in cells (22). On the other hand, zinc may block iron-sulfur cluster biogenesis, thus producing apo-form inactive proteins in cells. To delineate the two possibilities, we added ZnSO_4_ (200 μM) to the E. coli *zntA zntR* mutant cells in LB medium before and after recombinant iron-sulfur protein was expressed.

**FIG 3:**
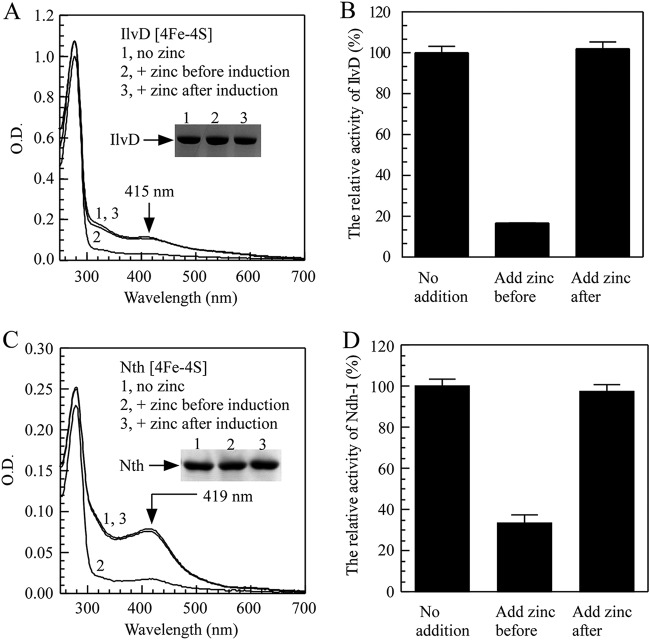
Excess zinc indirectly disrupts iron-sulfur cluster assembly in proteins. (A) Inhibition of the [4Fe-4S] cluster assembly in recombinant IlvD in the *E. coli* cells by zinc. UV-visible absorption spectra of recombinant IlvD purified from *E. coli zntA zntR* double-mutant cells supplemented with 0 μM (spectrum 1) or 200 μM ZnSO_4_ grown in LB medium before (spectrum 2) and after (spectrum 3) protein was produced in the cells. The protein concentration of IlvD was 28 μM. The inset is a photograph of the SDS-PAGE gel of purified proteins. (B) Relative activity of purified IlvD from panel A. For the enzyme activity assay, 1 μM IlvD was used. The unit of IlvD enzyme activity referred to the production of keto acid (μM) per minute per micromolar IlvD. The relative activity of control sample was considered to be 100%, and the relative activity of other samples was obtained by dividing the control activity. (C) Inhibition of the [4Fe-4S] cluster assembly in recombinant endonuclease III (Nth) in the *E. coli* cells by zinc. UV-visible absorption spectra of recombinant endonuclease III (Nth) purified from *E. coli zntA zntR* double-mutant cells supplemented with 0 μM (spectrum 1) or 200 μM ZnSO_4_ grown in LB medium before (spectrum 2) and after (spectrum 3) protein was produced in the cells. The protein concentration of IlvD was 12 μM. The inset is a photograph of the SDS-PAGE gel of purified proteins. (D) Effect of zinc on NADH dehydrogenase I in the *E. coli* cells. Inverted membrane vesicles (10 μl) were added to 290 μl reaction solution containing Tris (20 mM, pH 8.0), NaCl (200 mM), and deamino-NADH (50 μM). NADH dehydrogenase I activity was measured by monitoring the oxidation of deamino-NADH at 340 nm (extinction coefficient, 6.22 mM^−1 ^cm^−1^) at room temperature. The unit of NADH dehydrogenase I enzyme activity referred to the reduction of substrate (micromolar) per minute per OD at 600 nm. The relative activity of control sample was considered to be 100%, and the relative activity of other samples was obtained by dividing the control activity. The results represent average ± standard deviation from three independent experiments.

In the experiment, recombinant dihydroxy-acid dehydratase (IlvD), which contains a [4Fe-4S] cluster for its enzyme activity in the branched-chain amino acid biosynthesis pathway (49), was expressed in the E. coli *zntA zntR* mutant cells grown in LB medium with or without ZnSO_4_ (200 μM). IlvD was then purified from the cells. Figure 3A shows that the addition of ZnSO_4_ (200 μM) to LB medium before recombinant IlvD was expressed largely eliminated iron-sulfur clusters in the protein. However, the addition of zinc to LB medium after IlvD was expressed did not significantly affect iron-sulfur clusters in the protein. The enzyme activity measurements further showed that zinc blocked iron-sulfur cluster assembly without affecting the preassembled iron-sulfur clusters in IlvD in the E. coli cells (Fig. 3B).

The recombinant endonuclease III (Nth), a DNA repair enzyme which hosts a stable [4Fe-4S] cluster (50), was also investigated. Figure 3C shows that while the addition of ZnSO_4_ (200 μM) to LB medium before endonuclease III was expressed in the E. coli *zntA zntR* mutant cells largely prevented iron-sulfur cluster assembly in the protein, the addition of ZnSO_4_ (200 μM) to LB medium after endonuclease III was expressed did not significantly affect the preassembled iron-sulfur cluster in endonuclease III.

To further explore the inhibition of zinc overload on endogenous iron-sulfur cluster biogenesis, we measured the activity of the native NADH dehydrogenase I in the E. coli cells. Figure 3D shows that while the addition of ZnSO_4_ (200 μM) to LB medium followed by 5 h of growth of the E. coli *zntA zntR* mutant cells significantly decreased the enzyme activity, the addition of ZnSO_4_ (200 μM) to LB medium after 5 h of cell growth did not affect the enzyme activity of the NADH dehydrogenase I in the cells.

In E. coli, in addition to the housekeeping *iscSUA-hscBA-fdx-iscX* iron-sulfur gene cluster assembly system, there is another stress-inducible *sufABCDSE* system. Since increased expression of the gene cluster *sufABCDSE* is an indication of the iron-sulfur cluster assembly deﬁciency in E. coli cells (51, 52), we also analyzed the expression of the *sufA* operon in the E. coli *zntA zntR* mutant cells in response to ZnSO_4_ in LB medium and found that expression of the *sufA* operon was indeed induced by ZnSO_4_ treatment (Fig. S2B), suggesting that zinc has a general inhibitory effect on iron-sulfur cluster biogenesis in E. coli cells.

Taken together, the results suggested that ZnSO_4_ (200 μM) inhibits iron-sulfur cluster biogenesis without affecting the preassembled iron-sulfur clusters in proteins in E. coli cells.

### IscU, IscA, and ferredoxin are the major zinc targets among the housekeeping iron-sulfur cluster assembly machinery.

The proteins encoded by *iscSUA-hscBA-fdx-iscX* represent the housekeeping iron-sulfur cluster biogenesis machinery in E. coli cells. If zinc inhibits iron-sulfur cluster biogenesis, it is possible that zinc may directly interact with iron-sulfur cluster assembly proteins. To test this idea, we expressed each protein encoded by the gene cluster *iscSUA-hscBA-fdx-iscX* in the E. coli *zntA zntR* mutant cells grown in LB medium supplemented or not with ZnSO_4_ (200 μM). Purified proteins were then subjected to the UV-visible absorption measurements and metal content analyses.

**FIG 4:**
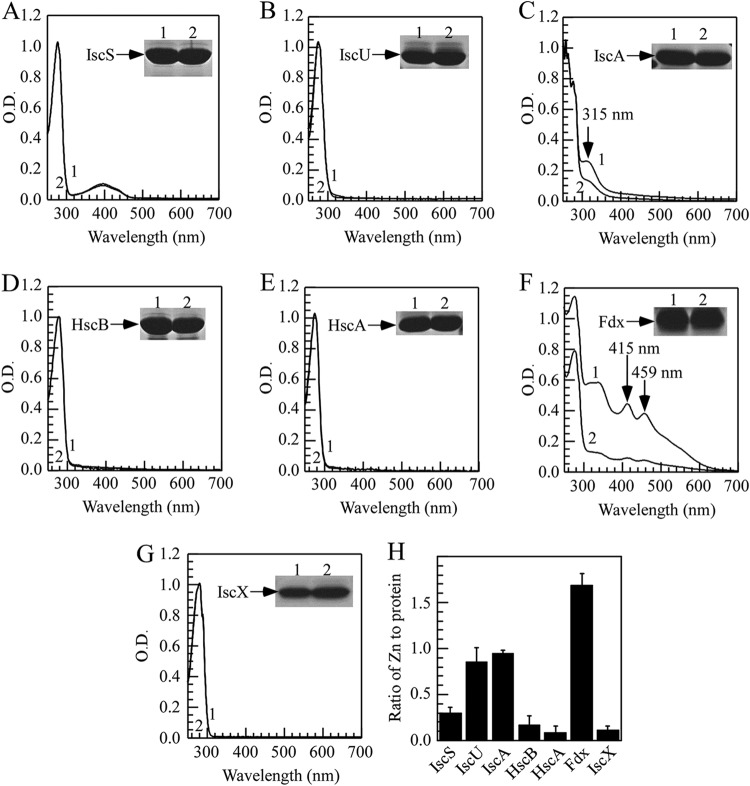
IscU, IscA, and ferredoxin are the major zinc binding proteins among the iron-sulfur cluster assembly proteins. Each protein encoded by the gene cluster *iscSUA-hscBA-fdx-iscX* was expressed in the *E. coli zntA zntR* double-mutant cells grown in LB medium supplemented or not with 200 μM ZnSO_4_. Proteins were purified from the cells and subjected to UV-visible absorption measurements. (A) IscS. (B) IscU. (C) IscA. (D) HscB. (E) HscA. (F) Ferredoxin. (G) IscX. In each panel, spectrum 1 is without ZnSO_4_ in LB medium, and spectrum 2 is with 200 μM ZnSO_4_ in LB medium. The inset in each panel is a photograph of the SDS-PAGE gel of purified proteins. The results are representatives of three independent protein preparations. (H) The zinc content of the iron-sulfur cluster assembly proteins encoded by the gene cluster *iscSUA-hscBA-fdx-iscX* purified from the *E. coli zntA zntR* double-mutant cells grown in LB medium supplemented with 200 μM ZnSO_4_. The results represent the average ± standard deviation from three independent experiments.

Figure 4 shows that the addition of ZnSO_4_ (200 μM) to LB medium had no effect on the UV-visible absorption spectra of IscS (Fig. 4A), IscU (Fig. 4B), HscB (Fig. 4D), HscA (Fig. 4E), and IscX (Fig. 4G) expressed in the E. coli *zntA zntR* mutant cells. On the other hand, the addition of ZnSO_4_ (200 μM) to LB medium significantly decreased the iron binding peak at 315 nm of IscA (Fig. 4C) and the iron-sulfur cluster binding peaks at 415 nm and 459 nm of ferredoxin (Fig. 4F) expressed in the E. coli *zntA zntR* mutant cells, suggesting that zinc overload may block the iron binding in IscA and the iron-sulfur cluster binding in ferredoxin. The zinc content measurements in purified proteins (Fig. 4H) showed that IscU, IscA, and ferredoxin proteins contained 0.85 ± 0.16, 0.94 ± 0.04, and 1.69 ± 0.12 zinc atoms per protein monomer (*n* = 3), respectively. The stoichiometry of zinc binding in IscU is consistent with previous studies showing that each IscU monomer contains one zinc atom (24). On the other hand, other iron-sulfur cluster assembly proteins had only very little or no zinc binding (Fig. 4H). Thus, IscA, IscU, and ferredoxin are the major targets of zinc overload in the E. coli *zntA zntR* mutant cells.

### The conserved cysteine residues in IscA, IscU, and ferredoxin are required for their zinc binding activity.

To explore the zinc binding sites of IscU, IscA, and ferredoxin, we constructed an IscU mutant (IscU-3M) in which three cysteine residues (Cys-37, Cys-63, and Cys-106) were replaced with serine, an IscA mutant (IscA-3M) in which three cysteine residues (Cys-35, Cys-99, and Cys-101) were replaced with serine (48), and a ferredoxin mutant (Fdx-4M) in which four cysteine residues for binding the [2Fe-2S] cluster (Cys 42, Cys 48, Cys 51, and Cys 87) were replaced with serine. Wild-type IscU, IscA, and ferredoxin and their mutants (IscU-3M, IscA-3M, and ferredoxin-4M, respectively) were then expressed in the E. coli *zntA zntR* mutant cells grown in LB medium supplemented with increasing concentrations of ZnSO_4_ (0 to 400 μM). Each protein was then purified from the E. coli cells.

**FIG 5:**
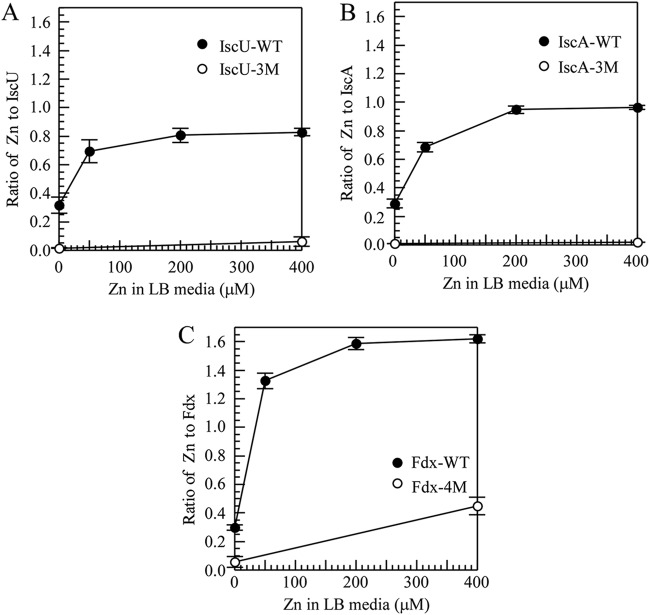
The conserved cysteine residues are required for the zinc binding activity for IscA, IscU, and ferredoxin. (A) Zinc binding activity of IscU in *E. coli zntA zntR* double-mutant cells in LB medium. Wild-type IscU and IscU mutant (IscU-3M) were expressed in *E. coli zntA zntR* double-mutant cells grown in LB medium supplemented with the indicated concentrations of ZnSO_4_. Proteins were purified from *E. coli* cells and subjected to the zinc content analyses. Zinc content in purified IscU was plotted as a function of the ZnSO_4_ concentration in LB medium. Closed circles, wild-type IscU; open circles, IscU-3M mutant. (B) Zinc binding activity of IscA in *E. coli zntA zntR* double-mutant cells in LB medium. Wild-type IscA and IscA mutant (IscA-3M) were expressed in *E. coli zntA zntR* double-mutant cells grown in LB medium supplemented with indicated concentrations of ZnSO_4_. Proteins were purified from *E. coli* cells and subject to zinc content analysis. Zinc content in purified IscA was plotted as a function of the ZnSO_4_ concentration in LB medium. Closed circles, wild-type IscA; open circles, IscA-3M mutant. (C) Zinc binding activity of ferredoxin in *E. coli zntA zntR* double-mutant cells in LB medium. Wild-type Fdx and an Fdx mutant (Fdx-4M) were expressed in *E. coli zntA zntR* double-mutant cells grown in LB medium supplemented with indicated concentrations of ZnSO_4_. Proteins were purified from *E. coli* cells and subject to zinc content analysis. Zinc content in purified Fdx was plotted as a function of the ZnSO_4_ concentration in LB medium. Closed circles, wild-type Fdx; open circles, Fdx-4M mutant. The results represent the average ± standard deviation from three independent experiments.

Figure 5 shows that zinc binding in IscU, IscA, and ferredoxin was gradually increased in the E. coli *zntA zntR* mutant cells as the concentration of ZnSO_4_ in LB medium was increased. In contrast, the mutant proteins (IscU-3M, IscA-3M, and Fdx-4M) expressed in the E. coli *zntA zntR* mutant cells had very little or no zinc binding even after 400 μM ZnSO_4_ was added to LB medium. Thus, IscU, IscA, and ferredoxin have specific zinc binding activity, and the conserved cysteine residues in IscU, IscA, and ferredoxin are essential for their zinc binding activity.

### Zinc overload emulates the phenotype of an E. coli mutant with the deletion of IscU, IscA, and ferredoxin.

**FIG 6:**
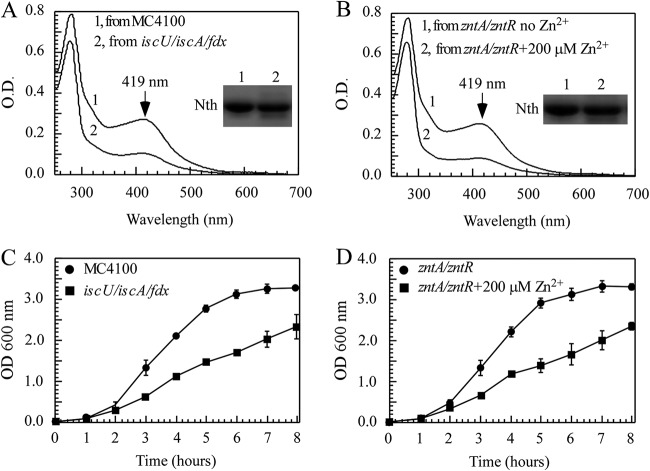
Excess zinc emulates the phenotype of an *E. coli* mutant with deletion of IscA, IscU, and ferredoxin. (A) UV-visible absorption spectra of recombinant endonuclease III (Nth) purified from *E. coli* MC4100 (spectrum 1) and *iscU iscA fdx* mutant (spectrum 2) in LB medium under aerobic growth conditions. (B) UV-visible absorption spectra of recombinant endonuclease III (Nth) purified from *E. coli zntA zntR* mutant cells (spectrum 1) supplemented with 200 μM ZnSO_4_ (spectrum 2) in LB medium under aerobic growth conditions. Insets in panels A and B are photographs of an SDS-PAGE gel of purified proteins. (C) Growth curve of *E. coli iscU iscA fdx* mutant (closed squares) and wild type *E. coli* MC4100 (closed circles) in LB medium under aerobic growth conditions. The results represent the average ± standard deviation from three independent experiments. (D) Growth curve of *E. coli zntA zntR* mutant supplemented (closed squares) or not (closed circles) with 200 μM ZnSO_4_ in LB medium under aerobic growth conditions. The results represent the average ± standard deviation from three independent experiments.

If IscU, IscA, and ferredoxin are the major targets of zinc overload in the E. coli cells, the deletion of these genes would emulate the effects of zinc overload on iron-sulfur cluster biogenesis. To test this idea, we deleted the genes encoding IscU, IscA, and ferredoxin to produce an E. coli *iscU iscA fdx* mutant. Figure 6A shows that the deletion of IscU, IscA, and ferredoxin decreased the iron-sulfur cluster assembly in endonuclease III, which was similar to the inhibition of the iron-sulfur cluster assembly in endonuclease III in the E. coli *zntA zntR* mutant cells grown in LB medium supplemented with 200 μM ZnSO_4_ (Fig. 6B). We also measured the cell growth of the E. coli mutant with the deletion of IscA, IscU, and ferredoxin and found that those deletions resulted in slow growth (Fig. 6C), which was also similar to that of the E. coli *zntA zntR* mutant cells grown in LB medium supplemented with 200 μM ZnSO_4_ (Fig. 6D). Thus, zinc overload in E. coli cells appears to emulate the phenotype of the E. coli mutant cells with deletion of the iron-sulfur cluster assembly proteins IscU, IscA, and ferredoxin.

## DISCUSSION

In this study, we report that zinc overload in the E. coli *zntA zntR* mutant cells inhibits iron-sulfur cluster biogenesis without affecting the preassembled iron-sulfur clusters in proteins or proteins without iron-sulfur clusters. Additional studies show that among the housekeeping iron-sulfur cluster assembly proteins encoded by gene cluster *iscSUA-hscBA-fdx-iscX* in E. coli, IscU, IscA, and ferredoxin have strong zinc binding activity in E. coli cells, and the conserved cysteine residues in the proteins are essential for their zinc binding activity. The deletion of IscU, IscA, and ferredoxin in E. coli cells appears to emulate the zinc overload-mediated inhibition of iron-sulfur cluster biogenesis and the slow-growth phenotype of the E. coli *zntA zntR* mutant cells. The results suggest that zinc overload inhibits iron-sulfur cluster biogenesis by specifically targeting IscU, IscA, and ferredoxin in cells.

Iron-sulfur clusters in proteins are generally vulnerable to reactive oxygen species (51) and nitric oxide (53). Recently, it has also been shown that iron-sulfur clusters in dehydratases are sensitive to copper (54), zinc, and silver (22). Here, we find that iron-sulfur proteins, including fumarases A and B, endonuclease III, biotin synthase, and endogenous NADH dehydrogenase I, are readily inactivated by zinc overload in E. coli cells. On the other hand, the preassembled iron-sulfur clusters in proteins are not disrupted by zinc overload in the cells. Thus, zinc overload appears to target iron-sulfur cluster biogenesis instead of disrupting iron-sulfur clusters in proteins in cells. This notion of zinc toxicity is analogous to that of copper toxicity, which also inhibits iron-sulfur cluster biogenesis without affecting preassembled iron-sulfur clusters in cells (48, 55).

One finding of this study is that zinc can effectively inhibit the enzyme activity of NADH dehydrogenase I in the E. coli *zntA zntR* double-mutant cells. This is partially in agreement with the previous observation that zinc inhibits the respiratory chain by both respiratory oxidases (56, 57) and NADH dehydrogenase (57) in E. coli. However, we also find that the activity of preexisted NADH dehydrogenase I is not affected significantly by zinc in E. coli *zntA zntR* double-mutant cells. The apparent contradiction with the previous report that zinc inhibits NADH dehydratase directly *in vitro* (57) is likely due to the different experimental conditions. A possible consideration is that the activity of NADH dehydrogenase is sensitive to zinc by determination *in vitro*. In our experiment, prior to the determination of NADH dehydratase I activity, most of the residual zinc after treatment was removed by washing cells twice with buffer. When we measured the activity of NADH dehydratase I, there was little or no zinc in the reaction solution. The activity of the NADH dehydratase I should be unaffected as long as the complex and the bound iron-sulfur clusters are completely assembled and not destroyed by zinc treated in living cells. Altogether, zinc may not only inhibit the activity of NADH oxidase and quinol oxidases directly but also decrease NADH dehydratase I activity by blocking the iron-sulfur cluster assembly in the enzyme complex in E. coli cells.

The effects of high zinc content on wild-type E. coli cells have been extensively investigated by transcriptomics (13), proteomics (58) and metalloproteomics (59) approaches. In response to the elevated intracellular zinc content, the cells will express multiple proteins, including ZraP, a putative zinc storage protein (59), a major zinc efflux system, ZntA, a P-type ATPase transporter, and a transcription factor, ZntR (40), among others. When ZntA and ZntR are deleted, the E. coli mutant cells accumulate intracellular zinc content, resulting in zinc overload. Because zinc and the iron-sulfur cluster have similar ligand coordination in proteins, it is conceivable that zinc overloading in cells may compete with iron or iron-sulfur cluster binding in the proteins and inhibit iron-sulfur cluster biogenesis in cells. With the exception of IscS, the major iron-sulfur cluster assembly proteins have either iron or iron-sulfur cluster binding sites. Here, we found that zinc overload in the E. coli *zntA zntR* mutant cells inhibits iron-sulfur cluster biogenesis by specifically binding to IscU, IscA, and ferredoxin. The zinc binding in the iron-sulfur cluster assembly protein IscU has previously been reported (60), which is consistent with our results. It may be envisioned that zinc overload forces IscU to bind zinc, which would prevent IscU from assembling iron-sulfur clusters. Zinc binding in IscA has not been previously reported. In the crystal structure, Cys-99 and Cys-101 of IscA are not visible, likely because of their flexible structure (61, 62). Nevertheless, it has been postulated that IscA may form a cysteine pocket with Cys-99 and Cys-101, which are responsible for binding iron (63) and facilitate the binding of other transition metal ions, such as copper or zinc. Our previous study showed that excess copper in E. coli cells does lead to copper binding in IscA under aerobic (48) and anaerobic (55) growth conditions and inhibits iron-sulfur cluster assembly. Here, we found that zinc overload in E. coli cells results in zinc binding in IscA and blocks iron-sulfur cluster biogenesis. Similarly, ferredoxin hosts a [2Fe-2S] cluster via four cysteine residues (Cys-42, Cys-48, Cys-51, and Cys-87) (64), and zinc overload in E. coli cells leads to zinc binding in ferredoxin. Since mutations of the conserved cysteine residues in IscU, IscA, and ferredoxin almost abolish the zinc binding activity of the proteins, these residues are critical for both iron/iron-sulfur clusters and zinc binding in the proteins. We propose that zinc overload in cells results in zinc binding in IscU, IscA, and ferredoxin and inhibits iron-sulfur cluster biogenesis in E. coli cells.

Since iron-sulfur proteins are involved in diverse physiological processes ranging from energy metabolism to DNA repair and replication (65), the inhibition of iron-sulfur cluster biogenesis by zinc will have a broad impact on diverse cellular functions. It should be pointed out that iron-sulfur proteins are also the targets of cobalt (66) and copper (48, 54, 55, 67, 68) toxicity. Thus, iron-sulfur cluster biogenesis could be the primary target of heavy-metal toxicity in cells.

## MATERIALS AND METHODS

### Gene knockout in E. coli cells.

ZntA and ZntR, two major proteins regulating intracellular zinc homeostasis, were deleted from wild-type E. coli (MC4100) following procedures described previously (69). The constructed E. coli *zntA zntR* mutant cells grow normally in LB medium but become hypersensitive to zinc in the medium as reported by Binet and Poole (41). Genes encoding IscU, IscA, and ferredoxin were also deleted from wild-type E. coli cells (MC4100). The gene deletion was confirmed by PCR. All primers for the gene deletion and confirmation were synthesized by TaKaRa Co. (Dalian, China).

### Protein expression and purification.

Genes encoding fumarases A, B, and C from E. coli were amplified using PCR and cloned to an expression plasmid, pBAD, as described previously (55). The plasmids expressing E. coli IscS, IscU, IscA, HscB, HscA, ferredoxin, IscX, dihydroxy-acid dehydratase (IlvD), endonuclease III (Nth), and biotin synthetase (BioB) were previously prepared. Each plasmid was introduced into the E. coli *zntA zntR* mutant cells. Cells containing the expression plasmid were grown to an optical density at 600 nm (OD_600_) of 0.6. ZnSO_4_ was then added to Luria-Bertani (LB) medium 10 min before recombinant protein was induced with 0.02% arabinose at 37°C for 4 h with aeration. Cells were harvested and washed twice with protein purification buffer (NaCl [500 mM], Tris [20 mM, pH 8.0]). Proteins were purified as described previously (55). The purity of purified protein was judged from SDS-PAGE, followed by the Coomassie blue staining. The concentration of purified protein was determined from the absorption peak at 280 nm using the published extinction coefficients.

### Site-directed mutagenesis.

The plasmid pBAD-IscA-3M was previously constructed. The IscU-3M (C37/63/106S) and ferredoxin-4M (C42/48/51/87S) mutants were constructed by the site-directed mutagenesis. Each of the conserved cysteine residues in the proteins was replaced with serine. PCR primers used in this work are shown in Table 2, and specific mutations were confirmed by direct sequencing.

**TABLE 2:** PCR primers used in this work

Primer	Sequence^*a*^
IscU-C37S-1	ATGGTGGGGGCACCGGCCTCTGGCGACGTGATG
IscU-C37S-2	GAGGCCGGTGCCCCCACCATGCCGCTG
IscU-C63S-1	CGTTTTAAAACTTACGGCTCTGGTTCCGCTAT
IscU-C63S-2	GAGCCGTAAGTTTTAAAACGCGCGTC
IscU-C106S-1	CCGCCGGTGAAAATTCACTCTTCTATTCTGG
IscU-C106S-2	GAGTGAATTTTCACCGGCGGCAGTT
Fdx-C42S-1	TGAACACGCCTCTGAAAAATCCTG
Fdx-C42S-2	GAGGCGTGTTCAATCTCGATACCG
Fdx-C48,51S-1	GCTTCTACCACCTCTCACTGCATCGTTCGT
Fdx-C48,51S-2	AGAGGTGGTAGAAGCACAGGATTTTTC
Fdx-C87S-1	AGCCGTTTAAGCTCTCAGGCGCGCGTTAC
Fdx-C87S-2	AGAGCTTAAACGGCTTTCCGGCTCCAG

a The underlined bases indicate mutation sites.

### Enzyme activity assays for fumarases, dihydroxy-acid dehydratase, NADH dehydrogenase I, and cysteine desulfurase.

The activities of purified fumarases A, B, and C were measured by monitoring the reaction product (fumaric acid) in a reaction mixture containing 50 mM sodium phosphate (pH 7.4) and 50 mM substrate (malate) at 250 nm using an extinction coefficient of 1.48 cm^−1 ^mM^−1^ (55). For dihydroxy-acid dehydratase, activity was measured using dl-2, 3-dihydroxy-isovalerate as the substrate. In the assay, 10 μl purified IlvD was added to 390 μl preincubated solution containing 50 mM Tris (pH 8.0) and 10 mM substrate. The reaction product (keto acids) was monitored at 240 nm using an extinction coefficient of 0.19 cm^−1 ^mM^−1^. NADH dehydrogenase I activity of E. coli cells was measured following procedures described previously, with some modifications (48). Briefly, the inverted membrane vesicles of E. coli cells were prepared by passing the cells through low-temperature ultrahigh-pressure continuous flow cell disrupter (JN-3000 Plus) once. Inverted membrane vesicles were added to the reaction solution containing 20 mM Tris (pH 8.0), NaCl (200 mM), deamino-NADH (100 μM), sodium azide (400 mM), and plumbagin (400 μM). The NADH dehydrogenase I activity was determined by measuring the oxidation of deamino-NADH at 340 nm (ε = 6.22 mM^−1 ^cm^−1^) at room temperature. In this situation, deamino-NADH as a specific substrate for NADH dehydrogenase I provides electrons (47), sodium azide inhibits the terminal cytochrome oxidases (52), and plumbagin abstracts the electrons directly from the NADH dehydrogenase I (59). The cysteine desulfurase activity of E. coli IscS was measured by incubating IscS with dithiothreitol (2 mM) and l-cysteine (0.1 mM) at 37°C. The amount of sulfide produced by IscS in the solution was measured according to Siegel’s method (70).

### Metal content analyses.

Total zinc content in protein samples was determined using the zinc indicator PAR [4-(2-pyridylazo)-resorcinol]. The iron content of protein samples was measured according to Fischer’s method (71). Zinc and iron contents in protein samples were also analyzed by the inductively coupled plasma-emission spectrometry (ICP-MS). The results from the two methods were very similar to each other.

For total zinc content of E. coli, cells was also determined by ICP-MS. Particularly, the E. coli *zntA zntR* mutant and its parental wild-type strain MC4100 cells were grown in LB medium supplemented or not with 200 μM ZnSO_4_ at 37°C under aerobic conditions. Cells were harvested from 50 ml LB medium by centrifugation when the OD at 600 nm of the cells reached 0.6. The cell pellet was washed twice with 50 ml of 170 mM NaCl, 20 mM Tris-HCl (pH 8.0), and 2 mM EDTA, resuspended in 5 ml of 170 mM NaCl, and transferred to a microwave digestion vessel with 4 ml nitric acid (35% [vol/vol], Shanghai Yiqian Technology Co. Ltd.) added. The vessel was sealed and placed in the microwave chamber. The following steps were run: the temperature was ramped to 120°C, held for 1 min, ramped to 160°C, held for 6 min, ramped to 180°C, held for 20 min, and reduced to room temperature over 30 min. Once digestion was complete, the samples were diluted using deionized water. The zinc content of above-mentioned samples was determined by ICP-MS.

## Supplementary Material

Supplemental file 1
