# Supplementary material for: Zinc Toxicity and Iron-Sulfur Cluster Biogenesis in Escherichia coli
Source: Appl Environ Microbiol. 2019 Apr 18;85(9):e01967-18. doi: 10.1128/AEM.01967-18 (PMC6495748; doi:10.1128/AEM.01967-18)
Supplement: Supplemental file 1 [file AEM.01967-18-s0001.pdf]

Supplemental Figures

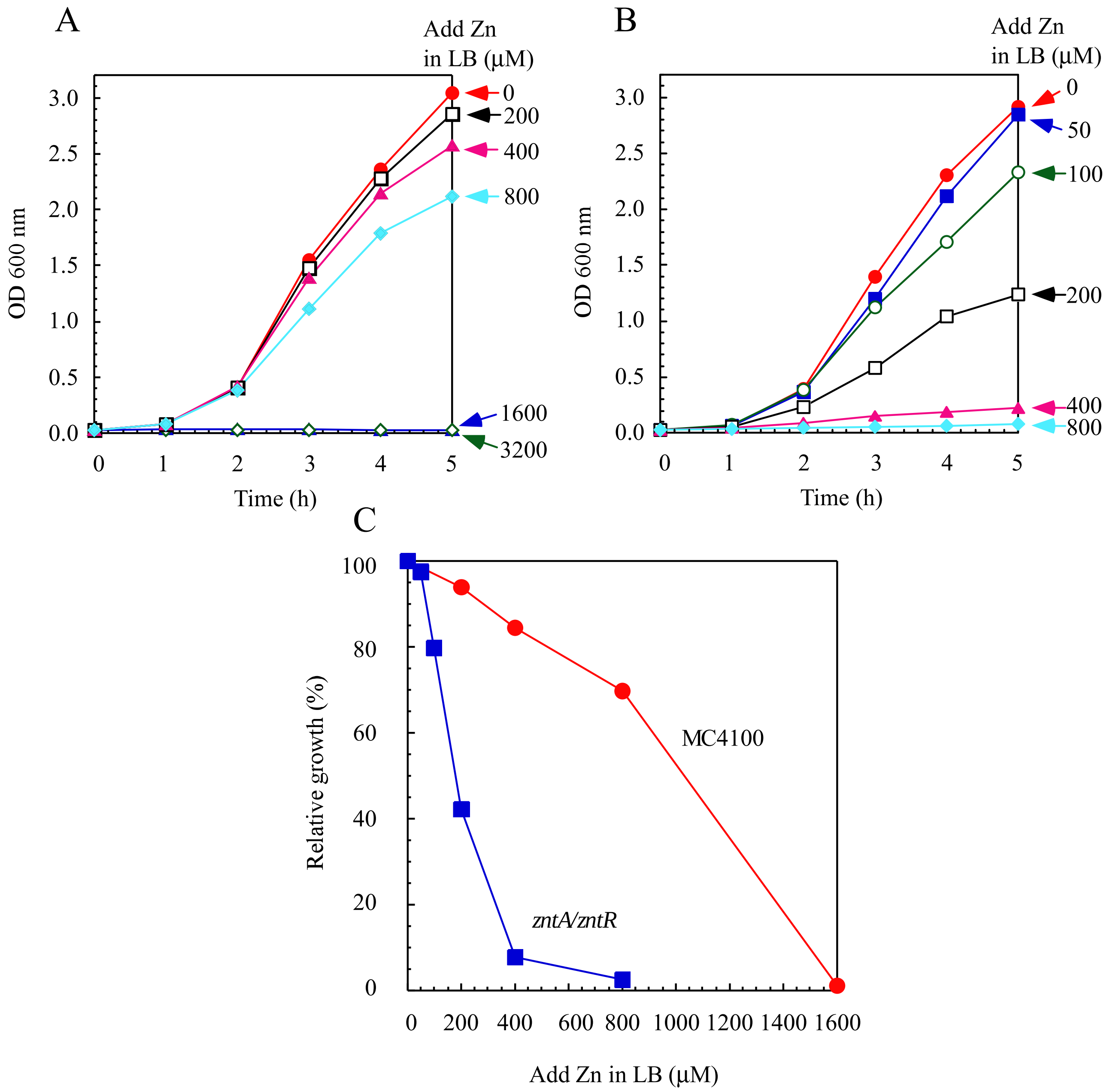

Supplemental Figure 1. The inhibition effect of zinc to *E. coli zntA/zntR* double mutant.

A) Growth curve of wild type of MC4100 *E. coli* cells grown in LB medium supplemented with 0, 200, 400, 800, 1600 and 3200 μM $ZnSO_4$. **B)** Growth curve of *E. coli zntA/zntR* double mutant cells grown in LB medium supplemented with 0, 50, 100, 200, 400 and 800 μM $ZnSO_4$. **C)** Relative growth of *E. coli zntA/zntR* double mutant cells (closed squares) and wild type MC4100 (closed circles) from panel A and B were plotted as a function of the $ZnSO_4$ concentration in LB medium. The 100% cell growth represented the cell density of O.D. at 600 nm after 5 h at 37°C with aeration in LB medium without $ZnSO_4$ treatment.

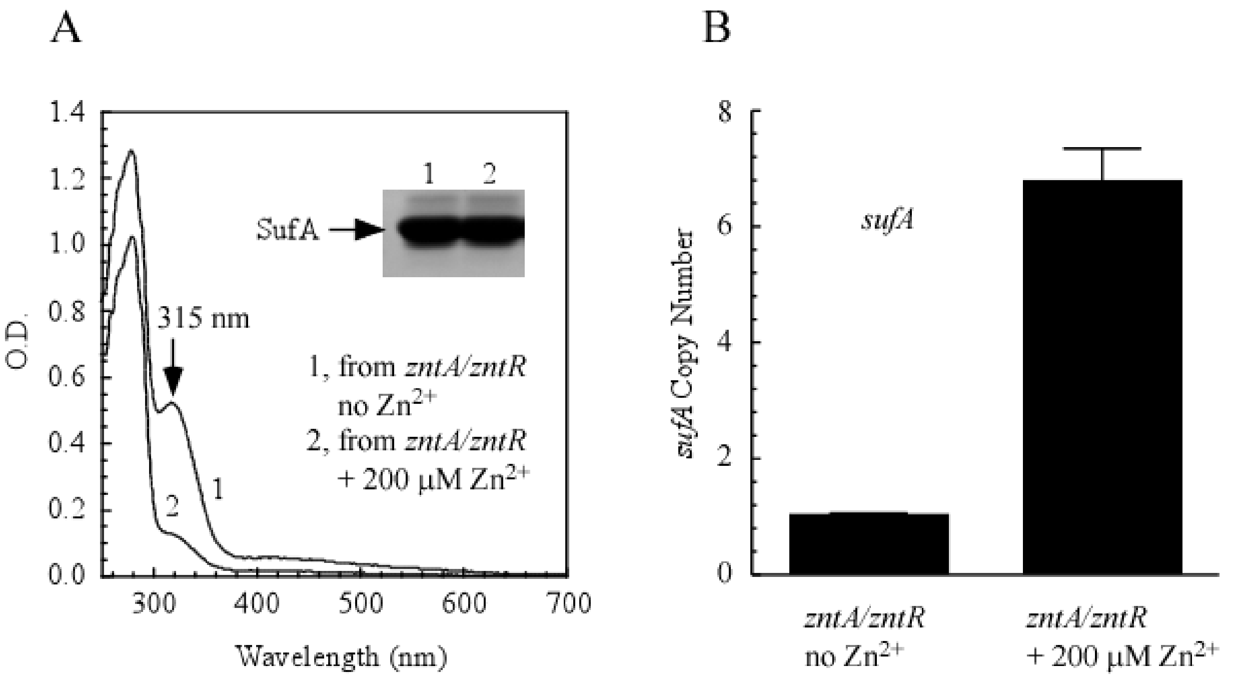

Supplemental Figure 2. Effect of zinc overload on SufA protein and *sufA* gene in *E. coli* cells.
A) UV-visible absorption spectra of recombinant SufA purified form *E. coli zntA/zntR* mutant cells grown in LB medium supplemented with (spectrum 2) or without (spectrum 1) 200 µM $ZnSO_4$. The absorption peak at 315 nm indicates the iron binding in SufA. The zinc content of purified SufA was determined using the ICP- MS/MS (Agilent 8800). The zinc binding ratio to SufA purified form *E. coli zntA/zntR* mutant cells grown in LB medium supplemented with 200 µM $ZnSO_4$ is 0.77±0.01. And the zinc binding ratio to the control sample is lower than 0.30. Insert in the panel is a photograph of SDS-PAGE gel of purified proteins.
B) Quantitative RT-PCR analyses of *sufA* gene transcript in *E. coli zntA/zntR* mutant cells grown in LB medium supplemented with or without 200 µM $ZnSO_4$. Total RNA was isolated from *E. coli zntA/zntR* mutant cells grown in LB medium supplemented with or without 200 µM $ZnSO_4$ for quantitative RT-PCR analyses. The gene *mdoG* was used as an internal reference of RT-PCR. The results were averages ± standard deviations from three independent experiments.

Materials and Methods

Quantitative RT-PCR analyses. Overnight *E. coli zntA/zntR* mutant cultures were 1:50 diluted in fresh LB medium supplemented with or without 200 µM $ZnSO_4$, and grown in LB medium at 37°C with aeration until O.D. at 600 nm of 0.6. Total RNA was extracted from *E. coli* cells using the Trizol solution, followed by removal of genomic DNA using gDNA Eraser (Clontech co). Primers for quantitative RT-PCR were listed below. Transcript of *E. coli* gene *mdoG* was used as a reference for

RT-PCR analyses (1).
sufA-1: 5'-TGGCTATGTGCTCGACAGTGTTAG-3'; *sufA*-2: 5'-CTTCACGAACGAAATCGACTTC-3'; *mdoG*-1: 5'-TAACTATCGTCCGGAGTTGCAC-3'; *mdoG*-2: 5'-TTCCATGGAGAAGCTGCTGA-3'.

1. **Heng SS, Chan OY, Keng BM, Ling MH.** 2011. Glucan Biosynthesis Protein G Is a Suitable Reference Gene in Escherichia coli K-12. ISRN microbiology **2011:**469053.
